# Ferulic Acid Attenuates Seizure Severity and Enhances Valproate and Carbamazepine Seizure Preventing Efficacy by Regulating Hippocampal Interleukin-1β Level and Antioxidant Capacity in Mice

**DOI:** 10.1155/omcl/8832818

**Published:** 2025-10-10

**Authors:** Elaheh Taheri, Majid Hassanpourezatti

**Affiliations:** Department of Biology, Basic Sciences School, Shahed University, Tehran, Iran

**Keywords:** carbamazepine, ferulic acid, interleukin-1β, maximal electroshock seizure, mice, sodium valproate, total antioxidant capacity

## Abstract

Epilepsy is a neurological brain disease characterized by multiple seizures with short intervals and becomes drug-resistant when it causes oxidative stress and inflammation in the brain. Ferulic acid (FA) is a plant phenolic substance with antioxidant, anti-inflammatory, and neuroprotective effects that is used in the treatment of neurodegenerative diseases in traditional medicine. This study evaluated the effect of FA (20 or 80 mg/kg) alone and then FA (20 mg/kg) combined with valproate (VPA) or carbamazepine (CBZ) on maximal electroshock-induced seizures (MESs) in mice associated with interleukin-1β (IL-1β) concentrations and total antioxidant capacity (TAC) assessment in the hippocampus. Male NMRI mice (weight 25–30 g) were injected intraperitoneally with saline (1 mL/kg), FA (20 or 80 mg/kg), diazepam (D) (20 mg/kg), VPA (200 mg/kg), CBZ (10 mg/kg), FA (20 mg/kg) + VPA (200 mg/kg), and FA (20 mg/kg) + CBZ (10 mg/kg) before application of MES. Duration of tonic hind limb extension (HLE), and chimney climbing time and grip-strength were also recorded. Then, mice scarified and hippocampus removed and homogenized. The level of IL-1β and TAC were determined. FA (20 and 80 mg/kg) significantly reduced the HLE duration and prevented seizure-induced hippocampal IL-1β increase and antioxidant capacity decrease. In addition, FA (20 mg/kg) enhanced the anticonvulsant efficacy of VPA and CBZ by regulating hippocampal IL-1β and antioxidant capacity. The finding suggest that FA possessed anticonvulsant effect and improved VPA and CBZ efficacy by regulating of IL-1β and antioxidant capacity in the hippocampus.

## 1. Introduction

Clinically, epilepsy is characterized by frequent and unpredictable seizures. The disease ranks high among the known neurological diseases worldwide, according to a report by the World Health Organization (WHO) [1, 2]. Approximately 1% of people worldwide and 2% of the Iranian population suffer from epilepsy [3, 4]. Although the mechanisms are unknown, it has been suggested that transient abnormal neuronal activity following local elevation of cytokines and oxidative stress mediators in the hippocampus may be a trigger for tonic-clonic seizures [5, 6]. In support of this theory, reports have demonstrated an increase in concentrations of IL-1β and its receptors in the hippocampus of patients with temporal lobe epilepsy [7, 8]. According to existing reports, increased levels of IL-1β in the hippocampus are one of the factors that cause a rapid decrease in GABA-A receptor-dependent chloride current, which in turn exacerbates the abnormal excitability of hippocampal neurons, and this abnormal activity can act as a trigger for the occurrence of epilepsy throughout the brain [9–11]. IL-1β can also stimulate the production of reactive oxygen species (ROS) through direct effects or indirectly by activating the enzyme NADPH oxidase in neurons [12].

Despite the wide variety of anticonvulsant drugs, unfortunately, the effectiveness of these drugs has been significantly reduced due to the emergence of drug-resistance in epileptic patients. Therefore, monotherapy is not sufficient to prevent and suppress tonic-clonic seizures, and the use of complementary drugs along with anticonvulsant drugs have been used to address this problem [13]. In such a situation, a practical solution is to use active plant-derived compounds as supplements to anticonvulsant drugs.

The maximal electroshock-induced seizure (MES) is a widely used and well-known model for inducing epileptic seizures through widespread activation of brain neural circuits. In this method, an alternating electric current is applied to the murine brain through wires connected to the ears and is used for preclinical evaluation of compounds that have potential for preventive or therapeutic use in this type of seizure. Animal showed seizures similar to those of human generalized tonic-clonic seizures [14]. It is worth noting that increased expression levels of some interleukins and decreased endogenous antioxidant reserves in the hippocampus are suggested factors for the exacerbation of epileptic seizures and the cause of drug-resistant epilepsy [15]. Therefore, administration of compound can reduce IL1-β levels and saved redox balance in the hippocampus may help to normal function of ion channels and prevent from seizure [16].

Increasing investigations have revealed that plant phenolic acids possess preventing and treating effect on epileptic seizures owing to their rich anti-inflammatory, antioxidant, and neuroprotective potentials [17, 18]. In addition, some phenolic acids have shown an additive effect on the antiepileptic drug actions [19, 20]. Ferulic acid (FA) is one of the phenolic acid that widely found in Iranian edible and medicinal plants such as Asteraceae family [21, 22]. It possesses a wide spectrum of health beneficial activities, including antioxidant and anti-inflammatory activity [23–25]. Previous evidence has shown that plant anti-inflammatory ingredients could improve efficacy of some anticonvulsant drugs [11]. Modern pharmacological evidence suggests that FA is the predominant phytochemical component in medicinal plants that have been known to have anticonvulsant effects in traditional medicine [26]. In another study, FA administration also showed antiepileptic efficacy against kindling model of epilepsy [27]. Further experimental studies have demonstrated FA neuroprotective and anticonvulsant potential through modulation of the nitric oxide pathway and the upregulation of GABA-B1 receptor expression [28]. Furthermore, a phenolic acid derivative has shown an anticonvulsant activity against maximal electroshock seizure [29]. On the other hand, administration of plant derived materials with valproate (VPA) and carbamazepine (CBZ) caused a synergistic effect on tonic-clonic seizures by reducing neuroinflammation and oxidative stress in CNS [30, 31]. In this line, FA supplementation improved anticonvulsive and neuroprotective capacities of vinpocetine, a voltage-gated sodium channel inhibitor [32, 33]. Thereby, its administration may prevent seizure, neuronal damage caused by production of cytokines, and oxidative stress mediators and potentiation of anticonvulsant drugs.

Given the unique history of FA presented above, this study aimed to evaluate the effect of pretreatment with FA or its coadministration with VPA and CBZ on MES-tonic-clonic seizures in mice. Moreover, the possible molecular mechanisms of these treatments were explored by measuring IL-1β level and total antioxidant capacity (TAC) in the hippocampus. Additionally, the safety of these treatments was evaluated by the chimney and muscle strength tests.

## 2. Materials and Methods

### 2.1. Chemicals and Reagents

FA (trans-4-hydroxy-3-methoxycinnamic acid), a mixture of 99% isomers, was purchased from Sigma–Aldrich (CAS Number 537-98-4). Diazepam (D) was received as a gift from Caspian Tamin Company, Iran. Sodium VPA was received as a gift from Rouz Darou Pharmaceutical Co. (Tehran, Iran). CBZ was received as a gift from Amin Pharmaceutical Company (Tehran, Iran). Mice interleukin-1β (IL-1β) ELISA kits was procured from Karmania Pars Gene Company, Iran. Total antioxidant assay kit was procured from Navand Salamat Company, Iran.

### 2.2. Animals and Ethical Approval

Adult male NMRI mice, weighing 25–30 g at the time of the experiment, were purchased from Pasteur Institute of Iran, Tehran, Iran. All mice were housed in standard laboratory conditions (22 ± 2°C and relative humidity of 55% ± 5%), and provided water and food available ad libitum. Experiments were conducted between 9AM and 2PM. Animals were housed in a ventilated room under a 12/12 h light/dark cycle.

Each experimental group consisted of 10 male mice, assigned to the group randomly. Each mouse was used only once. This study was conducted according to the National Institutes of Health Guide for the Use of Laboratory Animals (NIH Publication Number 23-80), 1996 amendment. The proposal for this research has been approved by the Ethics Committee of Shahed University with the code IR.SHAHED.REC.1402.037. All efforts were made to minimize animal suffering and to limit the number of animal used.

### 2.3. Acute Toxicity Testing With Chimney Test and Muscle Strength

The chimney test is conducted to quantify potential acute adverse effects caused by FA, VPA, and CBZ alone or after combination therapy on motor performance of mice [34]. The climbing time of animals backwards up in a vertical cylinder with a length of 30 cm and a diameter of 3 cm were recorded and compared among groups. The inability of mice to climb the chimney within 60 s was considered as impaired motor coordination [35]. The grip strength test is used to evaluate the effects of drugs and MES on muscle strength in mice. The grip-strength apparatus is an isometric force transducer attached to a steel wire ring. During the experiment, the mice were placed on a table in front of a force measuring device. The mice were forced to grasp the ring with their front paws. The mice were then lifted by their tails from the surface of the test table and pulled until they released the ring. The force (in gram) applied to transducer by the mouse before losing the ring is recorded. The mean of three measurements were calculated for each animal [36].

### 2.4. MESs

The anticonvulsant effect of each treatment on convulsion were evaluated by a previously described method [37]. Mice were randomly divided into 11 groups (*n* = 10): control group, received normal saline for 4 days without seizure; MES group, treated with normal saline 1 mL/kg, i.p. for 4 days before MES application; positive control group, received 20 mg/kg, i.p. D once immediately before MES; FA groups, received 20 or 80 mg/kg, i.p. FA for 4 days without receiving MES; FA + MES groups, received FA at the doses 20 or 80 mg/kg, i.p. for 4 days before MES application; VPA group, received 200 mg/kg i.p. VPA once before MES, and CBZ group, received 10 mg/kg i.p. CBZ once before MES; FA + VPA group received FA 20 mg/kg, i.p. for 4 days, and single VPA 200 mg/kg i.p. injection before MES; and FA + CBZ group received FA 20 mg/kg, i.p. for 4 days, and single injection of CBZ 10 mg/kg, i.p. before MES. After electroshock application, the duration of tonic hind limb extension (HLE) was recorded for each mouse and averaged for each group [37]. In this study, ED_50_ dose of FA was administered in combination with anticonvulsants to simulate the clinical dose of FA for antioxidant medication, based on data from a pilot study at this research center [38].

Maximal electroconvulsive seizure was induced by an electrical stimulator (Borj Sanat Company, Tehran, Iran) by applying an alternating current (50 Hz, 25 mA, 0.2 s) through ear clip electrodes. Electrodes were moistened with normal saline before attachment.

### 2.5. Determination of IL-1β Levels, TAC, and Total Protein Concentration in the Hippocampus

Immediately after the end of the experiments, the animals were sacrificed by decapitation under deep ether anesthesia. The brains were removed and homogenized in 5 volumes of Tris HCl (w/v) solution (50 mM, pH 7.4, 4°C) on ice with a sonication (3 periods × 15 s). The clear supernatant obtained after 15 min centrifugation at 3000 rpm. The total protein concentration, IL-1β, and TAC levels of supernatant were determined [39–41].

### 2.6. Statistical Analysis

Data were presented as the mean ± standard error of the mean (SEM). Data were compared by means of a two-way ANOVA followed by the Bonferroni post-test. Statistical analysis was performed by using GraphPad Prism version 5.0 (GraphPad Software Corporation, La Jolla, CA, USA). The adopted level of significance selected was *p* < 0.05.

## 3. Results

### 3.1. FA Improves the Motor Coordination of MES-Exposed Mice in the Chimney Test, While It Had a Different Effect on VPA and CBZ

The effect of FA administration alone or in combination with VPA and CBZ on the motor coordination of mice in the chimney test is presented in Table 1. Our findings show that administration of both doses of FA alone had no significant effect on chimney escape time of mice. A significant increase in chimney escape time was observed in mice after exposure with MES. FA could prevent from this increase in chimney escape time in mice received MES seizure. Chimney escape time significantly improved in D (20 mg/kg) pretreated mice before MES application compared to saline pretreated one. Further, pretreatment with VPA (200 mg/kg) and CBZ (10 mg/kg) before MES application prevented from increase of chimney escape time. Finally, FA was able to significantly improve the efficacy of CBZ on escape time from the chimney test, while it did not show such an effect on VPA. Moreover, in this study, the mortality rate in MES experiments was much less than 10%.

### 3.2. FA Improves the Efficacy of VPA and CBZ on MES-Induced Reduction in Muscle Strength in Mice

**Figure 1:**
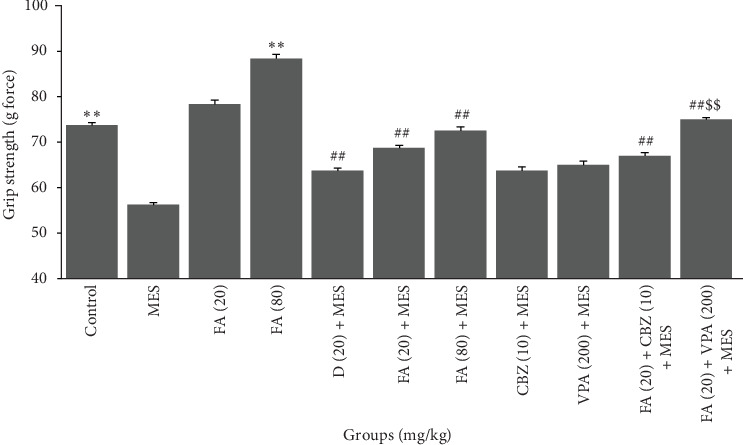
Effects of pretreatment with FA alone and in combination with VPA and CBZ on the grip strength test in mice. Mean grip strength of mice was recorded after treatment with FA in control and seizure induction. FA (80 mg/kg) significantly increased the mean grip strength in control animals. Moreover, FA at 20 and 80 mg/kg significantly increased the mean grip strength of mice exposure with MES. Finally, FA was only able to enhance the protective effect of CBZ against MES-induced reduction in grip strength in mice. Data are presented as mean ± SEM, maximal grip strengths in gram, assessing the neuromuscular strength in mice. Symbols for significant differences (*p*  < 0.01) are: *⁣*^*∗∗*^, between treated mice and normal control mice; ##, between treated mice and MES mice; $$ between combination therapy and anticonvulsant therapy alone.

Results of the ANOVA show statistically significant difference of muscular strength of mice among different groups (Figure 1). Comparing the effect of administering two doses of FA showed that only administration of 80 mg/kg could increase muscle strength in intact mice. A significant decrease in muscle strength was observed in mice following MES application. Administration of both doses of FA before MES have demonstrated protective action of it against MES caused muscle strength reduction similar to D (20 mg/kg). Although pretreatment of mice with VPA and CBZ alone could not significantly inhibit MES effect on muscle strength. Administration of FA with VPA only resulted in a significant increase in protection against muscle strength decline of mice after exposure to MES. (Figure 1).

### 3.3. Administration of FA Reduces the Severity of Seizure and Enhances the Anticonvulsant Action of VPA and CBZ

Effects of FA pretreatment on MES in mice are shown in Table 2. The present results showed administration of FA at both doses of 20 and 80 mg/kg, i.p. significantly decreased 26.9% and 30.1%, respectively) the duration of HLE following MES in mice as compared with saline-treated one. Pretreatment with D (20 mg/kg) caused 23% decrease in HLE duration as compared with saline-MES group. Injection of VPA (200 mg/kg) and CBZ (10 mg/kg), respectively, decreased HLE 20.60% and 23.8%, as compared with saline-MES group. The coadministration of FA (20 mg/kg) with VPA and CBZ reduced reduce HLE duration 44% and 35%, respectively, compared to saline-MES group.

### 3.4. FA Prevented the Seizure-Induced Decrease in Hippocampal Antioxidant Capacity and Enhanced the Antioxidant Protective Action of VPA and CBZ

**Figure 2:**
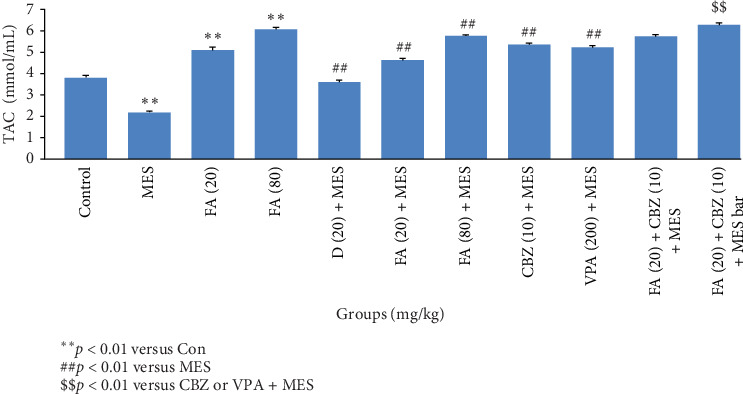
Changes in total antioxidant capacity (TAC) of mice brain in different treatment groups. Data were expressed as mean ± SEM (*n* = 10). *⁣*^*∗∗*^*p*  < 0.01 denotes a significant difference compared to the control group; ##*p*  < 0.01 denotes a significant difference compared to the electroshock group; $$*p*  < 0.01 denotes a significant difference compared to the single treatment. Comparison of the model group with each treated group was done through two-way ANOVA. CBZ, carbamazepine; D, diazepam; FA, ferulic acid; MES, maximal electroshock; VPA, sodium valproate.

Our findings showed that FA in a dose-dependent manner prevents from decrease of TAC in the hippocampus of intact mice (*p*  < 0.01). Mice hippocampal TAC significantly (*p*  < 0.01) reduced after exposure with MES compared to the control group. Pretreatment with D significantly (*p*  < 0.01) prevented the TAC reduction by MES. Administration of FA in a dose-dependent manner could prevent the seizure reducing effect on TAC level. The administration of VPA and CBZ also prevented from reduction of TAC in mice after convulsion and enhanced TAC level to a greater than its level in control mice. Administering FA with VPA had potentiate TAC in the hippocampus of mice exposure with MES, but its administration with CBZ did not lead to same effect (Figure 2).

### 3.5. FA Suppress Seizure- Induced Elevation of IL-1β and Enhanced VPA and CBZ Actions

**Figure 3:**
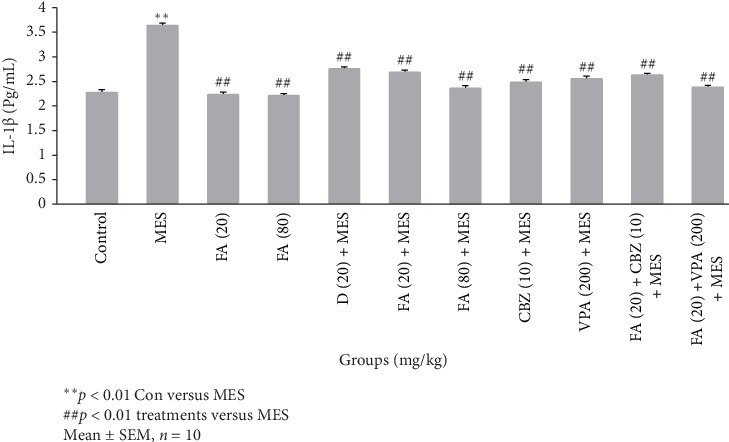
Change in the brain interleukin-1beta (IL-1β) expression level of mice in different groups. Data were expressed as mean ± SEM (*n* = 10). *⁣*^*∗∗*^*p*  < 0.01 electroshock group compared to control group; ##*p*  < 0.01 treatment groups compared to the electroshock group. Comparison of the model group with each treated group was done through two-way ANOVA. CBZ, carbamazepine; Con, control; D, diazepam; FA, ferulic acid; MES, maximal electroshock; VPA, sodium valproate.

As shown in Figure 3, MES caused a significant increase (*p*  < 0.01) in hippocampal IL-1β level compared to the control intact group. Pretreatment with FA in both doses of 20 and 80 mg/kg alone had no significant effect on the level of IL-1β in the hippocampus of intact control mice. D caused a significant (*p*  < 0.01) decrease in IL-1β levels in the hippocampus of mice receiving MES compared to saline pretreated mice. Administration of both doses of 20 and 80 mg/kg of FA in a dose-dependent manner (*p*  < 0.01) could suppress seizure increased IL-1β levels in the hippocampus of mice. Pretreatment with 200 mg/kg VPA and 10 mg/kg CBZ significantly (*p*  < 0.01) prevented from seizure induced increase of hippocampal IL-1β levels in mice. Administering 20 mg/kg of FA could not potentiate VPA and CBZ reducing effect on hippocampal IL-1β content.

## 4. Discussion

This study for first time investigated the anticonvulsant potential of FA against MES-induced tonic-clonic seizure and related neuroinflammatory and oxidative stress inducing effects. The result also indicated that anticonvulsant efficacy of FA was near to D. In addition, FA pretreatment has potentiating action on VPA and CBZ anticonvulsive action on this seizure model by inhibition of IL-1β level, and antioxidant capacity reduction in the hippocampus. The results indicated that suppression of neuroinflammation and oxidative stress in hippocampus by FA participates in direct anticonvulsant action and differently on VPA and CBZ seizure protection against tonic-clonic seizure.

Plant phenols are consistently employed for preventing action against different neurological diseases, including Alzheimer's disease owing to oxidative stress and neuroinflammation suppressing action with minimum side-effects on the body organs [42]. In support of our finding, a previous study indicated coadministration of plant phenolic compounds enhancing action on anticonvulsive property of VPA [43].

Other studies have also confirmed the antioxidant and anti-inflammatory properties of FA in various experimental and clinical studies [18, 44–46]. In addition, neuroprotective properties of FA have been proved by several preclinical studies [47–49]. Similarly, other phenolic compounds have shown antiepileptic activity through potentiation of endogenous antioxidative and anti-inflammatory mechanisms [44, 50, 51]. It is claimed that FA potential in treating various neurodegenerative diseases originates from its action on modulating multiple targets in CNS [52]. In this regards, FA has shown anti-inflammatory activity together with increasing the expression of GABA-B1 receptor [45]. Increase of GABA and acetylcholine level, concurrent with decrease of glutamate levels in the brain are other possible mechanisms for explanation of polyphenol efficacy against neurodegenerative disease [53]. Moreover, our results are in line with previous reports that has shown antiepileptic action of VPA and CBZ mediated by inhibition of IL-1β and regulation of the endogenous antioxidants in the brain [54, 55]. Indeed, it can say that suppressing the oxidative stress/cytokine activated signaling pathways can enhance efficacy of anticonvulsant drugs [56]. Further, antioxidants and anti-inflammatory agents have been shown direct anticonvulsant activity as well [57]. It has been found that administration of antioxidant agents not only prevents epilepsy but also reduces seizure-related CNS adverse effects [58].

In this study, electrically-induced seizure by MES model has been used as a well-studied method for inducing tonic-clonic seizure in mice through activation of cyclooxygenase-2, neuroinflammation, and oxidative stress imbalance in hippocampus [59, 60]. Pretreatment with FA reduced action on seizure, as demonstrated by its decreasing action on duration of HLE phase. This is a well-known parameter in electrical model of epileptic seizures correlated with ROS and cytokine levels in hippocampus [61]. It seems that neuronal hyperexcitability in the brain and behavioral symptoms of tonic-clonic seizures are directly associated with intensity of oxidative and inflammatory stress factor levels in the hippocampal neurons [62, 63]. Notably, tonic-clonic seizures lead to neurodegeneration in the hippocampus due to decreased antioxidants and increased cytokine production [64]. In consistent with this hypothesis, we show that an elevation of neuroinflammatory and oxidative stress factors in the hippocampus of animals are associated with duration of HLE induced by MES [65].

The results showed that FA can significantly reduce seizure duration and IL-1β concentrations, and prevent antioxidant capacity reduction in the hippocampus. Interestingly, researchers recently demonstrated that pretreatment with FA can prevent hippocampal neuron degeneration and cognitive outcomes in a genetic mouse model of Alzheimer's disease [66]. Also, FA, as a regulator of the MEK/ERK/p90RSK pathway, counteracts oxidative stress damage due to disruption of energy metabolism and has been able to reduce the destructive effects of focal ischemic conditions in the brain [67]. Another study revealed that FA can protect from hippocampus against neurodegenerative disease [68]. Therefore, our results were consistent with those of previous studies where MES received animals experienced oxidative stress and revealed a reduction in endogenous antioxidant capacity.

In addition, it has been reported that the anticonvulsant effects of VPA and CBZ decrease with time, due to oxidative stress mediated downregulation of GABA receptors in the brain [69, 70]. This is consistent with previous reports on the beneficial effects of FA in the treatment of drug-resistant epilepsy [71, 72]. Considering that compounds with antioxidant properties have been shown to be ideal supplements to enhance the efficacy of anticonvulsant drugs [73], it seems that FA could also be a good candidate for concomitant therapy with anticonvulsant drugs with the aim of better managing epileptic seizures. FA has also demonstrated a potential for stimulating the expression of endogenous antioxidant system mediators and masked the adverse effects of anticonvulsants [27, 74, 75]. The enhancing endogenous antioxidant capacity forms an important mechanism of phenolic compounds defense against oxidative insults in both glial cells and neurons [76]. Furthermore, FA combination therapy with VPA and CBZ reduced the hippocampal IL-1β concentration more than treatment with each one alone. Using repressors of the IL-1β receptor De Simoni et al. [77] concluded that the IL-β is directly involved in protecting the hippocampus from seizure.

Moreover, maximal electroshock seizure is one of the mice models for investigating the role of cytokines and oxidative stress process role in development of tonic-clonic seizures [78]. In line with other studies, the results showed that MES decrease antioxidant reserve and induced neuroinflammation in the hippocampus [79, 80]. Previous reports use MES as a model for the study of dysregulation of antioxidant/oxidative stress status and induction of inflammatory cytokines in the CNS [81–85]. Activation of these mechanisms in the hippocampus can lead to excessive stimulation of the neural networks of the brain and eventually epilepsy [86, 87]. So, decrease in IL-1β level and decrease of antioxidant reserve in the hippocampus are suggested as predominant mechanisms for epilepsy development [88].

The elevated level of IL-1β has been suggested as an underlying pathogenic mechanism for recurrence of seizure [89]. Antiepileptic drugs use also showed an increase in systemic oxidative stress, decrease of brain antioxidant capacity, and muscle performance in epileptic patients [90–92]. Interestingly, in the present study, it is uncovered that FA possessed protective effects on VPA and CBZ by attenuating above mentioned mechanisms. Literature reports on VPA and CBZ monotherapy are in line with our results and confirm that pretreatment with VPA and CBZ increased IL1-β and decreased antioxidant capacity [61, 93]. Therefore, it is confirmed that reduction of IL-1β and elevation of endogenous antioxidant capacity in the hippocampus may be preventing mechanisms for tonic-clonic seizures [94, 95]. In line with our findings, it is shown that supplementation with curcumin, a phenolic acid, improved anticonvulsants therapy efficacy [57]. As another example, it is shown that administration of isopentyl ferolate, an ester of FA, led to amelioration of convulsion [79]. In addition, it was reported that FA seizure reducing action associated with suppression of neuronal cell death, which supports our hypothesis that FA exerts its neuroprotective potential against seizure [96]. These findings are consistent with previous findings that the administration of phenolic compound not only prevent from seizure, but also enhance anticonvulsant drugs action [97–100].

Our reported findings are also consistent with reports showing that neuroprotective effect of FA/VPA and FA/CBZ combination therapy on seizure may relate to reduction of IL-1β and elevation endogenous antioxidant status [101]. Scientific evidence has confirmed the VPA and CBZ obstruct the entry of sodium ions into neurons, leading to decreased neuron excitability and firing rate and these mechanisms may have enhanced by antioxidants agents [102, 103]. So, the attenuating effect of FA in combination with above mentioned anticonvulsants on hippocampal IL-1β and antioxidant reserve confirmed existing reports about hippocampus as a target for action of agents such as FA, VPA, and CBZ for preventing of seizure by suppressing neuroinflammatory and oxidative stress factors [104–106].

## 5. Conclusion

In conclusion, the findings revealed that FA not only could be an anticonvulsant action through activation of central neuroprotective, anti-inflammatory and antioxidant mechanisms in MES model but also shown its potentiating action on antiepileptic efficacy of VPA and CBZ in mice. We also demonstrated certain safety aspects of FA, and its combination with anticonvulsants. Although, we observed the potential of FA to modulate hippocampal IL-1β, and antioxidant capacity alone and after combination therapy with VPA and CBZ; however, still, further experimentation is required to unveil its complete mechanism in seizure termination.

## Figures and Tables

**Table 1:** Effects of FA administration alone or in combination with CBZ and VPA on the escape time of mice in the chimney test.

Groups (mg/kg)	Time of escape chimney (s)
Control	8.7 ± 0.6
Saline-MES	14.38 ± 0.48*⁣*^*∗∗*^
FA (20)	8.17 ± 0.29
FA (80)	8 ± 0.5
Diazepam (20)-MES	10.75 ± 1.2*⁣*^*∗∗*^
FA (20)-MES	10.5 ± 0.5##
FA (80)-MES	9.37 ± 0.4##
CBZ (10)-MES	11.18 ± 1.02*⁣*^*∗∗*^
VPA (200)-MES	11.83 ± 1.63*⁣*^*∗∗*^
FA (20)-VPA (200)-MES	11.1 ± 0.74*⁣*^*∗∗*^
FA (20)-CPZ(10)-MES	10.9 ± 0.89$$

*Note:* Data were expressed as mean ± SEM (*n* = 10). Two-way ANOVA followed by Bonferroni post-testwas performed.

*⁣* ^*∗∗*^ *p*  < 0.01 denotes a significant difference compared with the control group.

##*p*  < 0.01 denotes a significant difference compared to the maximal electroshock group.

$$*p*  < 0.01 denotes a significant difference compared to single treatment with VPA or CBZ.

**Table 2:** Pretreatment with FA alone, and together with VPA and CBZ reduced the hind limb extension (HLE) duration in MES.

Treatments (mg/kg)	Duration (mean ± SEM) of hind limb extension (s)
Saline-MES	18.9 ± 0.57
Diazepam (20)-MES	14.5 ± 0.71*⁣*^*∗∗*^
FA (20)-MES	13.8 ± 0.5*⁣*^*∗∗*^
FA (80)-MES	13.2 ± 0.72*⁣*^*∗∗*^
CBZ (10)-MES	14.4 ± 0.5*⁣*^*∗∗*^
VPA (200)-MES	15.05 ± 0.07*⁣*^*∗∗*^
FA (20)-VPA (200)-MES	10.58 ± 0.99##
FA (20)-CBZ (10)-MES	12.25 ± 0.96##

*Note:* Data were expressed as mean ± SEM (*n* = 10). Two-way ANOVA followed by Bonferroni post-testwas performed.

*⁣* ^*∗∗*^ *p*  < 0.01 denotes a significant difference compared to the electroshock group.

##*p*  < 0.01 denotes a significant difference compared to single treatment group.

## Data Availability

The research data used to support the findings of this study are included within the article.
